# A Cross-Sectional Study on Subjective Fever Assessment in Children by Palpation: Are Fathers as Reliable as Mothers?

**DOI:** 10.1155/2020/3534267

**Published:** 2020-02-12

**Authors:** Ehud Rosenbloom, Crysta Balis, Dustin Jacobson, Melanie Conway, Ji Cheng, Eran Kozer

**Affiliations:** ^1^Division of Pediatric Emergency Medicine, McMaster Childrens Hospital, Hamilton, ON, Canada; ^2^Pediatric Emergency Department, Meir Medical Center, Kefar Saba, Israel; ^3^Department of Pediatrics, The Hospital for Sick Children, University of Toronto, Toronto, ON, Canada; ^4^Department of Clinical Epidemiology and Biostatistics, McMaster University, Hamilton, ON, Canada; ^5^St. Joseph's Healthcare Hamilton, Hamilton, ON, Canada; ^6^Pediatric Emergency Department, Shamir (Assaf Harofeh) Medical Center, Zerifin, Israel; ^7^Affiliated to the Sackler Faculty of Medicine, Tel Aviv University, Tel Aviv, Israel

## Abstract

**Background:**

Fever is common in pediatric patients. Often, parents rely solely on palpation when assessing their child's fever. The objective of the current study was to determine the accuracy of parents in detecting their child's fever by palpation.

**Methods:**

A prospective cross-sectional study was conducted at the emergency department (ED) of a tertiary pediatric hospital. Infants and children, 0–4 years of age, presenting to the ED with both parents were included. Parents were separately asked if their child had a fever and, if so, were asked to assess the temperature by palpation. A nurse obtained the rectal temperature. The primary outcome measure was the accuracy of fathers and mothers in detecting fever.

**Results:**

A total of 170 children with their parents were enrolled. The mean ages of the children, mothers, and fathers were 18.9 (SD 0.8) months, 31.1 (SD 6.4) years, and 33.7 (SD 6.9) years, respectively. No statistically significant difference was found between mothers and fathers in the ability to assess fever by palpation (OR 0.65, 95% CI 0.39,−1.08). Sensitivities for detecting fever by palpation for mothers and father were 86.4% and 88.2%, respectively (specificity among mothers: 54.2% and specificity among fathers: 43.1%). The overall negative and positive predictive values were 65.9% (95% CI 55%–75.7%) and 75.7% (95% CI 69.9%–80.8%), respectively.

**Conclusions:**

Mothers and fathers do not differ in their ability to accurately assess their child's fever by palpation. The low positive and negative predictive values indicate that if temperature was not measured, physicians cannot rely on parents' reports.

## 1. Introduction

Fever is a common presenting problem in pediatric patients in the office and emergency departments [1]. In many cases, parents do not measure the child's temperature and the assessment of fever often relies solely on palpation. In a study of Mexican immigrants, 40% of mothers never used a thermometer and more than 60% relied on palpation or visual inspection to determine fever in their child [2]. Tavers and colleagues [3] reported that parents with lower levels of education use thermometers less often to check for fever. Fever, particularly in young infants, is an important clinical sign that often requires prompt investigation and/or treatment. Numerous studies assessed the accuracy of tactile fever by a parent compared with the measured temperature [4–15].

Although some of these studies [4, 14, 15] reported a sensitivity in the range of 95% to 100%, a recent study [10] found a much lower sensitivity of only 63%. The reported specificity of mothers' palpation for detecting fever varies from 19% [15] and 23% [14] to 92% [4]. A systematic review published in 2008 [13] analyzed the results from 10 different studies. The pooled sensitivity was 89% and specificity was only 50%. However, there was a significant heterogeneity among the studies; furthermore, in all of these studies, only the mothers were studied.

Fathers and mothers may have different approaches when assessing their child's condition. For example, differences were found in parental response to pain [16].

There are no studies that investigated the reliability of tactile fever in fathers alone or in comparison with mothers. We hypothesized that there will be a significant difference between mothers and fathers in the ability to detect fever in their children. The objectives of the current study were to determine the accuracy of fathers and mothers in detecting their child's fever by palpation and to identify demographic factors that may be associated with the ability to identify fever by palpation.

## 2. Materials and Methods

A cross-sectional descriptive study was conducted at the emergency department (ED) of the McMaster University Children's Hospital, in Canada, from October 1^st^ 2011 to July 1^st^ 2013. The two study cohorts were father and mother defined as either biological parents or legal guardians of the children presented to the ED. Children aged 0–4 years in stable condition were included. Children were excluded if they presented without both parents, if their parents did not agree to rectal temperature measurement, or if the parents could not read and write English. This study was approved by the Research Ethic Board (REB) of McMaster University.

### 2.1. Study Protocol

After obtaining informed consent, parents were separated. Each parent was asked to palpate his/her child and to determine if the child had fever. If the answer was “Yes,” the parent was further asked whether it was high fever. The data collector observed the palpation event and documented the specific site chosen by the parent. The accuracy was later determined by comparing the parents' answers to the measures obtained from rectal temperatures (Sure Temp^R^ Plus 692, WelchAllyn, Skaneateles Falls NY) administrated by the healthcare professionals. Fever and high fever were defined as a temperature of 38.0°C or higher and 39.0°C or higher, respectively.

Other relevant information such as the basic demographics of the parents (age, level of education, employment status, etc.) and their preferred methods of measuring their children's temperatures was collected through self-administrated questionnaires.

### 2.2. Sample Size and Outcomes

We calculated that 120 pairs of parents are needed to detect a 15% difference between mothers and fathers, with a power of 80% and α of 0.05. For the purpose of adjusting for confounding variables, we added 50 pairs of parents to our final sample sizes, which allowed us to include up to 5 other variables in our final statistical model according to the rule of thumb of 5–10 events per variable.

The primary outcome measure was the accuracy of fathers and mothers in detecting the presence of fever. The secondary outcome was the ability of parents to detect high fever by palpation.

### 2.3. Statistical Analysis

Fathers' and mothers' demographic and other information were presented as percentages for categorical data and as mean and standard deviations (SD) for continuous data. The accuracy of parents detecting the presence of their children's fever or high fever using palpation was reported as sensitivity and specificity with their corresponding 95% confidence intervals (CI). Multiple logistic models were used to adjust for confounding variables that may affect the accuracy of fathers' and mothers' abilities to detect fever. Clustered robust standard error was used to adjust for potential clustering effect. The estimates from the logistic models were reported as odd ratios (ORs), the corresponding 95% CIs, and *p* value. The statistical significance was set as *p* equal to or less than 0.05. All analyses were conducted using STATA 13.1 (College Station, Texas, USA).

## 3. Results

Two hundred nine parents were approached for consent. Thirty-nine refused to consent mostly because of the need for rectal temperature measurement. Basic characteristics of those who signed the consent were not different from those who refused. The final data available for the analysis included 170 children, 170 mothers, and 168 fathers.

The mean ages of the children, mothers, and fathers were 18.9 ± 0.8 months, 31.1 ± 6.4 years, and 33.7 ± 6.9 years, respectively. Basic characteristics of the parent's demographics are presented in Table 1. Compared with fathers, mothers were more likely to believe that they could estimate fever by palpation (68.5% vs. 84.7%, respectively).

There were no differences in mothers' and fathers' abilities to accurately identify fever (74.1% among mothers and 72.6% among fathers) and high fever (63.1% among mothers and 59.2 among fathers) (Table 2).

After adjusting for the potential confounding or influential variables, fathers' abilities to correctly assess their children's fever was not statistically significantly different from the mothers' (OR (95% CI) = 0.65 (0.39–1.08), *p* = 0.099) (Table 3). However, the multiple logistic model showed that fathers' ability to decide whether their children had high fever was lower (OR (95% CI) = 0.56 (0.39–1.08), *p* = 0.050) (Table 4). The multiple logistic models also revealed that compared to parents working full time, the unemployed parents' ability to correctly assess their children's fever (OR (95% CI) = 0.44 (0.22, 0.88), *p* = 0.020) (Table 3) and high fever (OR (95% CI) = 0.48 (0.23, 0.90), *p* = 0.044) (Table 4) was significantly lower. The parents who brought their children to the ED for fever were able to more accurately assess their children's fever by palpation (OR (95% CI) = 1.96 (1.08–3.56), *p* = 0.027) (Table 3) compared to those who brought their children to the ED for other reasons.

Sensitivities for detecting fever among mothers and fathers were 86.4% and 88.2%, respectively. Specificities among mothers and fathers were 54.2% and 43.1%, respectively (Table 5). The overall negative predictive value was 65.9% (95% CI 55%–75.7%), and the positive predictive value was 75.7% (95% CI 69.9%–80.8%). Sensitivities for detecting high fever among mothers and fathers were 49.1%. Specificities among mothers and fathers were 74.6% and 67.1%, respectively (Table 6).

## 4. Discussion

In the current study, we found high sensitivity and low specificity for fever assessment by palpation. There was no difference between mothers and fathers in their ability to accurately assess their child's fever by palpation.

The sensitivity and specificity we found are in agreement with the finding of a meta-analysis published by Teng et al. [13]. Although sensitivities were high in both father and mother groups (>80%), the relatively low (65%) negative predictive value suggests that parents' palpation cannot be used as a screening tool to rule out fever. The specificities for both these groups were even lower (<50%) indicating that parents often overestimated the presence of fever. Future studies are needed in order to assess the proportion of children treated solely for fever based on parental palpation in developed countries. The impact of such practice on the utilization of healthcare services and emergency departments should also be investigated.

Palpating the child when he or she feels hot as a screening tool occurs in developing and developed countries. In developing countries, many parents do not use a thermometer and assess their child's fever by palpation [17]. In a survey of Latino parents in Baltimore, USA, 24% did not have thermometers at home [18]. It is therefore important for the medical staff to know how to interpret the parent's impression.

The relatively low negative and positive predictive values we found suggest that healthcare professionals should encourage parents to measure their child's temperature whenever their child is sick. These findings also indicate that physicians cannot rely on parent reports on fever if temperature was not measured.

Thermal sensation varies between genders [19, 20]. Based on these studies, we postulated that fathers will be less accurate than mothers in detecting fever in their offspring.

However, the results of the current study do not support this assumption.

An exception was the superior ability of mothers to correctly identify high fever than fathers. In our cohort, mothers were more likely to be the primary caretakers of the child. We expected that this would be another reason why mothers would be more accurate in estimating their child's temperature. Yet, we found that being the primary caretaker was not associated with better estimation of the child's fever. This finding is in agreement with the finding that unemployed parents were less likely to correctly detect fever in their offspring compared with employed parents. Both findings suggest that the time the parents spend with their offspring does not affect their ability to correctly assess their fever.

There were no differences between immigrants and native Canadians in the ability to correctly identify fever in their offspring, suggesting that cultural factors do not play major role in this issue.

We expected that the parent's age and the child's order in the family would have an effect on the parent's ability to accurately detect fever, as more experienced parents probably treated more febrile episodes of their children in the past. Our results do not support this assumption.

Despite the fact that the child's torso is covered with clothes or blankets, the site which parents used to palpate their child's fever did not affect their palpation accuracy.

Fever as a chief complaint was associated with more accurate palpation by the parents. This may be related to the fact that those parents probably measured their child's fever prior to coming to the ED. Another possible explanation is that those who presented with fever did so because they recognized it while those who did not actually did not recognize fever in their children.

This study has many strengths. First and most importantly, this is the first study to directly compare fathers with mothers in real-time in their assessment of their child's temperature by palpation. The parents were not aware how their spouse assessed the fever, and there was a very short delay between recruitment and parental assessment of fever and a triage temperature.

## 5. Limitations

The study was a single-center study, and the included patients presented to the ED with both parents. Selection bias could possibly result from these inclusion criteria.

We also do not know if the parents measured the child's temperature just before they came to the ED. Awareness of such measurement could also bias the results.

It is worth noting that not all parents consented to rectal temperatures taken for their infant; however, there were no significant differences in the basic characteristics between those who participated and those who refused to consent.

## 6. Conclusion

We found no difference between mothers and fathers in their ability to accurately palpate fever in their child. The positive and negative predictive values of parents' palpation to detect fever in their offspring are low. Consequently, we suggest that physicians and healthcare providers caring for young children should not rely on tactile fever.

## Figures and Tables

**Table 1:** Demographic and descriptive characteristics.

	Mother (*n* = 170) count (%)	Father (*n* = 168) count (%)	*p* value for difference
You are born			
** **Out of Canada	44 (25.9)	55 (32.7)	0.273
** **Unknown	0 (0.00)	1 (0.6)	
Level of education			
** **High school or less	44 (25.9)	58 (34.5)	0.217
** **College	92 (54.1)	76 (45.2)	
** **Graduate school	33 (19.4)	34 (20.0)	
** **Unknown	1 (0.6)	0 (0.0)	
Employment			
** **Unemployed	74 (43.5)	13 (7.7)	<0.001
** **Work less than 30 hrs	20 (11.8)	6 (3.6)	
** **Work 30–60 hrs	73 (42.9)	133 (79.2)	
** **Work 60–90 hrs	3 (1.8)	16 (9.5)	
Who spends more time with your child			
** **You	116 (68.2)	10 (6.0)	<0.001
** **Your spouse	6 (3.5)	109 (64.9)	
** **Equal	48 (28.2)	49 (29.2)	
Fever as reason of ED visit	106 (62.4)	110 (65.5)	0.527
Believe can estimate fever by palpation	144 (84.7)	115 (68.5)	0.002
Preferred method of measuring fever			
** **Thermometer	111 (65.3)	108 (64.3)	0.246
** **Palpation	36 (21.2)	44 (26.2)	
** **Both	1 (0.6)	3 (1.8)	
** **Other	22 (12.9)	13 (7.7)	
Site of palpation			
** **Forehead	110 (64.7)	132 (78.6)	0.029
** **Torso	14 (8.2)	10 (6.0)	
** **Both	26 (15.3)	14 (8.3)	
** **Other	19 (11.2)	9 (5.4)	
** **Unknown	1 (0.6)	3 (1.8)	
Number of children at home			
** **1	77 (45.3)	74 (44.1)	0.594
** **2	67 (39.4)	70 (41.7)	
** **3	18 (10.6)	12 (7.1)	
** **4 or more	8 (4.7)	11 (6.6)	
** **Unknown	0 (0.0)	1 (0.6)	
Parents' age (year)	Mean (SD) 31.1 (6.4)	Mean (SD) 33.7 (6.9)	<0.001
Children's age (month)	Mean (SD) 18.9 (0.9)		N/A

^*∗*^ *p* values were obtained by the chi-squared test for categorical data and two-group *t*-test for continuous outcomes.

**Table 2:** Assessment of fever by mothers and fathers.

	Mother	Father	*p* value for difference
	count/*n* (%)	count/*n* (%)	
Correctly estimated fever	126/170 (74.1)	122/168 (72.6)	0.755
Correctly estimated fever over 39°C	77/122 (63.1)	77/130 (59.2)	0.527

*p* values were obtained by the chi-squared test.

**Table 3:** Association between variables and identification of fever by palpation (*n* = 318).

Factors	OR	95% CI	*p* value
Father (ref = mother)	0.65	(0.39, 1.08)	0.099
Parent age (unit = 5 years)	0.85	(0.67, 1.10)	0.214
*Employment (ref* *=* *20–60 hrs)*			
Unemployed	0.44	(0.22, 0.88)	0.020
Less than 30 hrs	0.55	(0.21, 1.45)	0.229
60–90 hrs	0.62	(0.19, 2.05)	0.435
Fever as the reason of bringing child in	1.96	(1.08, 3.56)	0.027
Believe can estimate fever	0.55	(0.28, 1.11)	0.435
*Site of palpation (ref* *=* *forehead)*			
Torso	1.18	(0.46, 3.05)	0.727
Both	1.15	(0.49, 2.70)	0.740
Other spots	2.69	(0.80, 9.04)	0.110

ROC = 0.658.

**Table 4:** Association between variables and identification of fever over 39°C by palpation (*n* = 239).

Factors	OR	95% CI	*p* value
Father (ref = mother)	0.56	(0.31, 1.00)	0.05
Child's order in the family	1.14	(0.87, 1.49)	0.344
*Employment (ref* *=* *20–60 hrs)*			
Unemployed	0.48	(0.23, 0.90)	0.044
Less than 30 hrs	0.52	(0.17, 1.63)	0.262
60–90 hrs	1.14	(0.36, 2.62)	0.828
Believe can estimate fever	0.68	(0.37, 1.26)	0.223

ROC = 0.587; OR: odds ratio.

**Table 5:** Sensitivity and specificity for fever detection by palpation.

	Sensitivity (95% CI)(%)	Specificity (95% CI)(%)
Mother + father (*n* = 338)	86.4 (81.2, 90.7)	48.7 (39.4, 58.1)
Mother (*n* = 170)	84.7 (76.6, 90.8)	54.2 (40.8, 67.3)
Father (*n* = 168)	88.2 (80.6, 93.6)	43.1 (30.2, 56.8)

**Table 6:** Sensitivity and specificity for fever over 39°C detection by palpation.

	Sensitivity (95% CI)(%)	Specificity (95% CI)(%)
Mother + father (*n* = 252)	49.1 (39.5, 58.7)	70.7 (62.4, 78.1)
Mother (*n* = 122)	49.1 (35.4, 62.9)	74.6 (62.5, 84.5)
Father (*n* = 130)	49.1. (35.6, 62.7)	67.1 (55.1, 77.7)

95% CI: 95% confidence interval; *n*: sample size.

## Data Availability

The data used to support the findings of this study are available from the corresponding author upon request.
